# Cytotoxic and Immunochemical Properties of Viscumin Encapsulated in Polylactide Microparticles

**Published:** 2012

**Authors:** E.S. Kolotova, S.G. Egorova, A.A. Ramonova, S.E. Bogorodski, V.K. Popov, I.I. Agapov, M.P. Kirpichnikov

**Affiliations:** Biological Faculty, Lomonosov Moscow State University; Institute of Laser and Information Technologies, Russian Academy of Sciences; Shumakov Federal Research Centre of Transplantology and Artificial Organs

**Keywords:** biodegradable microparticles, viscumin, polylactide

## Abstract

Biodegradable polylactide microparticles with encapsulated cytotoxic protein viscumin were obtained via the ultrasound-assisted supercritical fluid technique. The size of the microparticles was 10–50 µM, as shown by electron microscopy. The time course of viscumin release from microparticles was studied using an immunoenzyme test system with anti-viscumin monoclonal antibodies. It was found that 99.91% of the cytotoxic protein was incorporated into polymer microparticles. Only 0.08% of the initially encapsulated viscumin was released from the microparticles following incubation for 120 h in a phosphate-buffered saline at neutral pH. Importantly, the method of ultrasonic dry supercritical fluid encapsulation failed to alter both the cytotoxic potency and the immunochemical properties of the encapsulated viscumin. Thus, this procedure can be used to generate biodegradable polylactide microparticles with encapsulated bioactive substances.

## INTRODUCTION

Viscumin, a ribosome-inactivating lectin, occurs in leaf extracts from the parasitic plant Common Mistletoe ( *Viscum album* ). Viscumin has a molecular weight of 60 kDa and consists of two subunits, A and B, which are linked via a disulfide bond [1, 2]. This protein has found widespread application in anti-tumor therapy [3–6]. Its efficacy can be enhanced by encapsulating viscumin into biodegradable polymer microparticles, thus ensuring its chemical and spatial stabilization, as well as a prolonged release of the protein into the surrounding tissues. Hence, the toxin will have a prolonged effect on tumor cells.

**Fig. 1:**
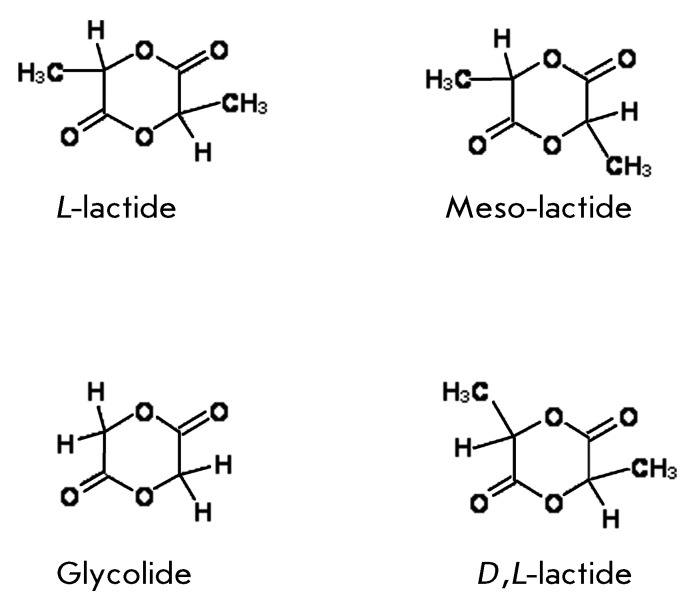
Cyclic dimers for the synthesis ofaliphatic polyesters.

Polylactides are a class of biodegradable polymers that belong to the homologous series of aliphatic polyesters that are finding increased application in biomedicine and pharmaceutics [7]. Polylactide is a polymer of lactic acid that contains asymmetric carbon atoms ( *Fig. 1)* and can easily form optically active cyclic dimers (lactides), which are capable of polymerizing via catalytic opening of 1,4-dioxane rings, similar to the polymerization of glycolides.

Polylactide contains methyl groups, and thus it is a more hydrophobic compound as compared to polyglycolide. Polyactide dissolves more easily in organic solvents. Since a monomer of lactic acid exists in two stereometric forms, four morphologically different polylactides can be synthesized: two stereo-regular polymers, poly( *D* -lactide) and poly( *L* -lactide); a polymerized blend of the *D* - and *L* -lactic acids – poly( *D,L* -lactide); and poly(meso-lactide) – *D* -and *L* -lactide blends. The polymers synthesized from the optically active *D* -or *L* -lactic acid only are polycrystalline, whereas the optically inactive poly( *D,L* -lactides) possess an amorphous structure. This fact is significant in their practical application, since the hydrolysis rate of these compounds (which determines their biodegradation kinetics in a living organism) is inversely proportional to their degree of crystallinity. Glycolide is a simpler compound that exists only in single form. The identity of the catalytic reaction of ring opening in glycolides and lactides enables copolymerization, yielding high-molecular-weight copolymers (polylactoglycolides); this fact considerably broadens the range of biodegradable synthetic materials that possess various biochemical and mechanical properties.

The ability of aliphatic polyesters to gradually biodegrade in the organism is used both to provide temporary protection to active molecules or drugs against rapid degradation by various enzymes and peptides and for the targeted delivery of these compounds to specific cells, tissues, and organs, as well as to control the release rate of these molecules from the polymer matrix, thus ensuring a more prolonged therapeutic effect [7]. For this purpose, a drug is encapsulated into a polymer carrier and is injected or introduced perorally into the organism [8, 9]. The method of dry supercritical fluid (SCF) encapsulation can be successfully used to incorporate various biologically active compounds (enzymes, peptides, proteins, and drugs) into polymer microparticles, the physicochemical and biological properties of which are almost completely retained [10–12]. This fact serves to differentiate SCF encapsulation from other methods, whose application assumes the use of high temperatures (up to 100°C and even higher) and toxic organic solvents; it is rather problematic to remove these solvents from the final product [10]. Supercritical carbon dioxide (sc-CO _2_ ) allows one to perform the encapsulation of bioactive components into various amorphous polymers without using liquid solvents at temperatures close to room temperature and under moderate pressure (the critical parameters for CO _2_ : *Т*_cr_  = 31°C, *Р*_cr_  = 7.4 МПа). Sc-CO _2_ can be easily and almost completely removed from the polymer by reducing pressure below the critical value [13].

The first model experiments devoted to the SCF encapsulation of viscumin into polylactide micromatrices were performed earlier [11]. It was revealed that the liberation time of viscumin can be controlled by varying the conditions of SCF encapsulation. This procedure actually enables the design of prolonged-action drugs with the desired release kinetics of the active substance from the polymer carrier.

Herein, the immunochemical and cytotoxic properties of viscumin, following its liberation from microparticles produced via the same procedure but with the use of ultrasound, were studied. Denser, fine particles (10–50 µm) were obtained using ultrasound.

## EXPERIMENTAL

Viscumin was kindly provided by Professor U. Pfüller (Institute of Phytochemistry, University of Witten/Herdecke, Germany). *D,L* -polylactide PURASORB PDL 02 (PURAC Biochem bv, Netherlands) with a molecular mass M _w _ ~ 20000 was used as an initial biodegradable polymer. Carbon dioxide of special purity grade (99.99%, Balashikha Oxygen Plant, Moscow oblast, Russia) was used without any additional purification. Dry phosphate buffered saline (PBS, Flow Laboratories, Great Britain), two-component reagent kit for the substrate mixture based on tetramethylbenzidine (TMB) for ELISA and the streptavidin–peroxidase conjugate (IMTEK, Russia); and polystyrene plates (Costar, USA) were also used. Monoclonal antibodies MNA4 and biotinylated MNA9 against various epitopes of the viscumin A-subunit were obtained earlier [14, 15]. The remaining reagents were purchased from Sigma-Aldrich Corporation (USA).

**Encapsulation of viscumin into polylactide microparticles**

The encapsulation of viscumin into the polylactide carrier was performed on an experimental setup similar to that described in [16]. The major difference from the previously used equipment [11] was the powerful ultrasonic action (18 ± 0.2 kHz, up to 1 kW that the polymer/viscumin system in sc-CO _2_ atmosphere was subjected to). This approach was implemented using a magnetostriction generator (MSG) with an acoustic concentrator and titanium inducer introduced into the high-pressure reaction chamber.

The formation of bioactive microparticles is attained via the following steps: The powder-like polylactide with a characteristic particle size of 100–200 µm (0.1 g) produced from preliminarily grinded initial polymer grains with a diameter of ~ 3÷4 mm and lyophilized viscumin powder (1 mg) were loaded into the high-pressure reaction chamber. The chamber was pressurized; CO _2_ at room temperature was fed until the pressure reached 5 MPa. The chamber heaters and nozzles were subsequently turned on. The temperature in the chamber was typically at 40 ^о^ С; the nozzle temperature varied from 40 to 80 ^о^ С. The pressure in the chamber increased with each rise in temperature. After the desired temperature was attained, the pressure in the chamber was brought to a pre-selected value of 10 ÷ 20 MPa. The MSG power control unit was then turned on; its power was varied within a range of 0.1–1.0 kW. The system was kept under these conditions for ~ 30 min to form the regime of SCF plasticization of the viscumin–polymer mixture in the reactor. A pulsed discharge of the plasticized mixture and carbon dioxide into the inlet chamber was subsequently performed using a pulsed valve, via a 0.5 mm diameter nozzle.

After the resulting product was stored in the inlet chamber under atmospheric conditions for 3 h (the time required for the complete removal of CO _2_ from polymer particles and the final hardening of the particles), the microparticles being collected were placed into 1.5 ml glass vials and were stored at a temperature of +4°C prior to the subsequent analysis.

**Scanning electron microscopy**

The morphology of the surface of the polymer microparticles with viscumin encapsulated via the sc-CO _2 _ technique was studied using scanning electron microscopy (SEM) on a LEO 1450 microscope (Carl Zeiss, Germany). A small amount of the powder under study was applied on a conducting (carbon) adhesive tape with a thin (~ 0.05–0.1 µm) gold film, which was coated onto it via plasma spraying and ensured the required conductivity.

**Investigation of the time course of viscumin liberation from polylactide microparticles**

A dry powder of viscumin-containing polylactide microparticles (16.5 mg) was suspended in 2 ml of PBS; the suspension was centrifuged for 10 min at 12,100 *g* ; and the supernatant was then sampled. The remaining polylactide particles were again suspended in 2 ml of PBS and then stirred on a rocker at 22°C. The supernatant was sampled, and the next PBS portion was added after 10, 30, 60, 120, 360, 1440, 2880, and 7200 min, respectively. The resulting samples were stored at +4°C.

**Analysis of the amount of viscumin liberated upon degradation of polylactide microparticles**

The amount of viscumin in the samples (i.e., in the supernatant sampled at different time points) was determined using the earlier described modified test system [14, 15]. Anti-viscumin monoclonal antibodies MNA4 at a concentration of 10 µg/ml in PBS were adsorbed onto a 96-well plate (100 µl per well). The antibodies were incubated for 24 h at +4°С and subsequently washed with a solution containing 20 mmol/l lactose and 0.05% Tween 20 in PBS. 100 µl of the buffer solution containing 0.1% of bovine serum albumin (BSA), 20 mmol/l lactose and 0.05% of Tween 20 in PBS were introduced into each well to block the vacant binding sites of the polystyrene surface. Following incubation for 1 h at 37°С, the antibodies were washed three times and 100 µl of the viscumin-containing samples under study in various dilutions were added to each well. Viscumin at different concentrations was used as a control sample. The incubation was performed for 1 h at 37°С. Biotin-labelled anti-viscumin monoclonal antibodies MNA9 at a concentration of 2 µg/µl were then applied. The following procedure included incubation for 1 h at 37°С, threefold washing, and incubation (1 h, 37°С) with the streptavidin–peroxidase conjugate and fivefold washing. The development was carried out for 20 min at 37°С using a TMB substrate buffer. The reaction was stopped by adding 50 µl of 10% sulfuric acid to each well. Colorimetric measurements were carried out on a Multiskan® PLUS-314 spectrophotometer at 450 nm.

The amount of viscumin incorporated into the microparticles was determined via solid-phase ELISA according to the above-described scheme. For this purpose, the sample of polylactide microparticles was completely hydrolyzed at 42 ^0^ С for 48 h (5 mg of the sample in 10 ml PBS).

**Evaluation of the cytotoxic properties of viscumin following its release from polylactide microparticles**

The cytotoxic activity of viscumin liberated from biopolymer microparticles was determined using the MTT assay according to the earlier described procedure [17, 18]. The lethal dose of toxin (viscumin) for 50% of the cells (LD _50_ ) was determined to assess the survival rate of the cells. Viscumin not subjected to encapsulation was used as the control. When calculating LD _50_ , the staining intensity of the cells cultured in the absence of a cytotoxic agent was assumed to be 100%. The results obtained in one of three typical experiments are presented (LD _50_  ± standard deviation).

## RESULTS AND DISCUSSION

**Fig. 2:**
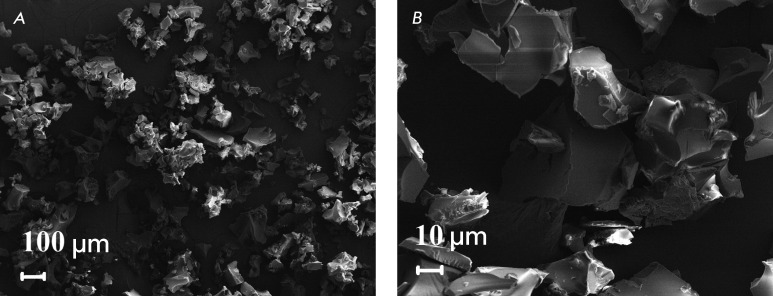
Electronic microimages of polylactide microparticles with incorporated viscumin. (A) – overview, (B) – detailed structure.

The typical SEM microimages of the experimental samples after their removal from the inlet chamber of the SCF setup are shown in *Fig. 2* . It is clear that the samples consist both of individual microparticles (10–50 µm) and particle agglomerates (up to 200 µm). Polylactide microparticles are dense bulk particles of irregular shape with an appreciably smooth surface.

The use of a powerful ultrasonic action made it possible not only to achieve more homogeneous stirring of the initial components (viscumin and polylactide), but to considerably reduce the viscosity of the polymer plasticized in sc-CO _2_ due to the introduction of additional acoustic energy to the system, as well. The combination of these factors resulted in a cardinal change in the regime of subsequent dispersion of the resulting mixture into the inlet chamber under atmospheric pressure. In turn, the morphology of the bioactive polymer structures being formed drastically changed.

Thus, polylactide fibrous matrices consisting of porous microparticles of irregular shape (50–200 µm) formed when the magnetic stirrer was used alone under the same conditions [11]. Meanwhile, the use of intensive acoustic action resulted in the formation of dense individual microparticles with the characteristic size varying from 10 to 50 µm.

The previously described test system based on the monoclonal anti-viscumin antibodies MNA4 and biotinylated MNA-9 [14, 15] was used to determine the amount of viscumin in the samples. The system enables the specific determination of viscumin; its sensitivity threshold is approximately 0.8 ng/ml. The total amount of viscumin in a 5 mg sample consisting of polylactide particles was assessed using this test system. The total amount of viscumin appeared to be equal to 50 µg, which corresponds to 1 wt % and represents the amount of viscumin subjected to encapsulation. Presumably, this attests to the fact that the protein antigenic structure after the encapsulation remained unchanged.

**Fig. 3:**
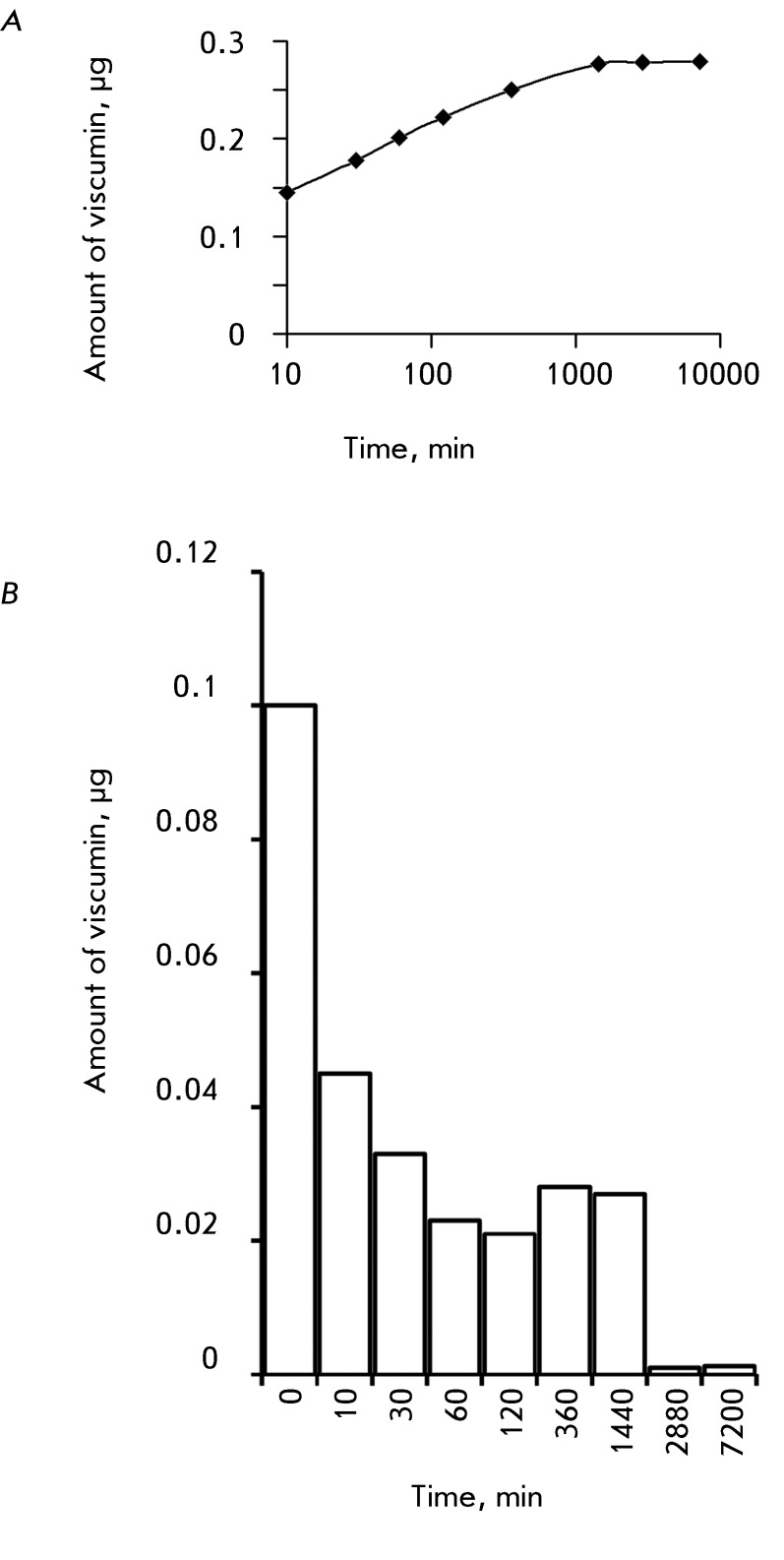
Kinetics of the release of viscumin from polylactide microparticles. (A) – the overall amount of viscumin in the supernatants under analysis. (B) – the amount of viscumin released from microparticles as a function of incubation time.

It is reasonable to assume that some nonencapsulated viscumin could remain on the microparticle surface. In order to remove the unbound viscumin, 16.5 mg of the microparticles was washed twice in PBS (time points 0 and 10 min). The amount of viscumin in these samples was 0.145 µg (i.e., 0.09% of the total amount of the toxin subjected to encapsulation). Thus, the amount of encapsulated viscumin was equal to 99.91%. *Figures 3A* and *3B* show the amount of viscumin liberated from polylactide microparticles during a period from 0 to 120 h.

It is clear from *Fig. 3B* that the toxin amount in supernatants decreased with incubation time. This fact indicates the slow degradation of polymer microparticles with the gradual release of viscumin. An increase in the amount of viscumin in supernatants at points 6 and 24 h can be explained by longer incubation, when a greater amount of toxin is liberated from the degraded polymer. In addition, starting at point 48 h, viscumin was released more slowly. This type of kinetics of toxin release may be linked with the structure of polylactide microparticles; i.e., inside a microparticle it is denser than outside. A total of 0.134 µg of viscumin was released from the polylactide matrix after 120 h (except for the amount of viscumin detected in the samples after they were washed twice), which is equal to 0.08% of the initially encapsulated protein.

Viscumin belongs to type II ribosome-inactivating proteins; it can be used to remove eukaryotic target cells [19–21]. It was demonstrated using the MTT assay that viscumin retains its cytotoxic activity after it is released from polylactide microparticles. The cytotoxic activity of viscumin in supernatants remained virtually the same as that of the untreated toxin: the concentration of the native viscumin causing the death of 50% of 3T3 cells (LD _50_ ) after 48 h was equal to 7 × 10 ^-12^  ± 3 × 10 ^-12^  М; LD _50_ of viscumin in the samples under analysis was equal to 7 × 10 ^-12^  ± 2 × 10 ^-12^  М.

It should be mentioned that the morphology of the surface and internal structure of the polymer microparticles being formed can be varied over an appreciably wide range (from highly porous to virtually monolithic) by changing the regimes of SCF encapsulation and sputtering of the plasticized polymer blend; this will have a determining effect on the kinetics of release of bioactive components from these particles. The results obtained clearly demonstrated this fact. Thus, the viscumin-containing polylactide structures in [11] were represented by agglomerates of porous particles (with a characteristic porosity coefficient of 20–25%) of irregular shape, as opposed to the dense microparticles with smooth surfaces, obtained in the present work ( *Figs.*   *3A* and *3B* ). Correspondingly, the kinetics of viscumin release from these structures under identical conditions (suspension in PBS, slow stirring on a rocker at 22°C; samples were collected after two washings and after 30, 60, 120, 360, 1440, 2880, and 7220 min) was cardinally different from that shown in *Fig. 3A* .

Viscumin can be used in anti-tumor therapy [22]. The application of biocompatible biodegradable systems containing slowly releasing viscumin has considerable potential. Drug introduction via injection into the area of tumor growth becomes less traumatic due to the reduction in the size of the biodegradable microparticles containing viscumin and/or another specific anti-tumor cytotoxin.

The ultrasound-assisted technique of dry supercritical fluid encapsulation does not affect the cytotoxic and immunochemical properties of the viscumin incorporated into polylactide microparticles. The designed encapsulation technique ensures the gradual release of the encapsulated toxin from microparticles, which can provide a more prolonged therapeutic effect. 

## Abbreviations

MLI: viscumin
RIP: ribosome inactivating protein
SCF: supercritical fluid
sc-CO_2_: supercritical carbon dioxide
PBS: phosphate buffered saline
TMB: tetramethylbenzidine
MSG: magnetostriction generator
SEM: scanning electron microscopy
BSA: bovine serum albumin
MTT: 3-(4,5-dimethylthiozolyl-2-yl)-2,5-diphenyltetrazolium bromide
LD_50_: lethal dose for 50% of the cells
